# Risk of developing a second primary cancer in male breast cancer survivors: a systematic review and meta-analysis

**DOI:** 10.1038/s41416-022-01940-1

**Published:** 2022-09-17

**Authors:** Isaac Allen, Hend Hassan, Eleni Sofianopoulou, Diana Eccles, Clare Turnbull, Marc Tischkowitz, Paul Pharoah, Antonis C. Antoniou

**Affiliations:** 1 Centre for Cancer Genetic Epidemiology, Department of Public Health and Primary Care, University of Cambridge, Cambridge, UK; 2 Department of Cancer Sciences, Faculty of Medicine, University of Southampton, Southampton, UK; 3 Translational Genetics Team, Division of Genetics and Epidemiology, Institute of Cancer Research, London, UK; 4 Department of Medical Genetics, National Institute for Health Research, Cambridge Biomedical Research Centre, University of Cambridge, Cambridge, UK

**Keywords:** Cancer epidemiology, Epidemiology, Risk factors, Breast cancer

## Abstract

**Background:**

With increasing survival after cancer diagnoses, second primary cancers (SPCs) are becoming more prevalent. We investigated the incidence and site of non-breast SPC risks following male breast cancer (BC).

**Methods:**

PubMed, Embase and Web of Science were systematically searched for studies reporting standardised incidence ratios (SIRs) for SPCs published by March 2022. Meta-analyses used the generic inverse-variance method, assuming a random-effects model. We evaluated SIRs for overall SPCs, site-specific risks, by age at BC onset, time since BC onset and geographic region. We assessed study quality using routine techniques.

**Results:**

Eight population-based retrospective cohort studies were identified. SIRs ranged from 1.05 to 2.17. The summary SIR estimate was 1.27 (95% CI: 1.03–1.56, *I*^2^: 86%), and there were increased colorectal (SIR: 1.29, 95% CI: 1.03–1.61), pancreatic (SIR: 1.64, 95% CI: 1.05–2.55) and thyroid (SIR: 5.58, 95% CI: 1.04–30.05) SPC risks. When an outlying study was excluded, the summary SIR for men diagnosed with BC before age 50 was 1.50 (95% CI: 1.21–1.85), significantly higher than men diagnosed at older ages (SIR: 1.14, 95% CI: 0.98–1.33).

**Conclusions:**

Male BC survivors are at elevated risks of developing second primary colorectal, pancreatic and thyroid cancers. The estimates may assist their clinical management and guide decisions on genetic testing.

## Background

Male breast cancer (BC) is rare, accounting for less than 1% of all BC cases [1, 2]. As a result, few studies have investigated the risks of second primary cancers (SPCs) following male BC [3–12]. SPCs in male BC survivors are a growing health problem. The age-standardised incidence rate of male BC rose by 40% between 1975 and 2015 [13], whereas the age-standardised male BC specific mortality rate decreased by 22.5% and 12.4% between 2002 and 2016 in the European Union and the USA, respectively [14]. Most clinical management guidelines for male BC are extrapolated from information on BC in postmenopausal women [2], so this review could better inform clinical management decisions regarding SPC prevention measures following male BC.

No systematic review of SPC risks following male BC has been performed since 2008 [15]. No meta-analysis of SPC risks following male BC has been carried out to date. We therefore aimed to conduct a significantly updated systematic review (SR) and a novel meta-analysis of SPC risks in male BC survivors. Our objective was to review the latest evidence regarding the risks of developing SPCs following a first invasive primary male BC. A further objective was to assess site-specific second cancer risks among studies that also investigated the overall SPCs risks. Our final objective was to evaluate the variability in non-breast SPC risks by confounding variables, such as patient characteristics.

## Methods

### Exposure, outcome and measures of association

The exposure was defined as a previous first primary invasive male BC, with no prior cancer history. The outcome was defined as a non-breast SPC.

To minimise misclassification of recurrences or metastases of the first BC as second primaries, SPCs were determined using one of two possible sets of guidelines: those given by the Surveillance, Epidemiology and End Results (SEER) programme [16], primarily used in North America [17] and those given by the International Association of Cancer Registries (IACR)/International Agency for Research on Cancer (IARC) [18, 19], used in all other regions [17]. An explicit statement that SPCs had been confirmed by a physician, with efforts made to differentiate SPCs from recurrences or metastases, was accepted if the guidelines used were unstated.

Second primary BC counts following a first BC are not comparable under the SEER and IACR/IARC guidelines, as observed in a 2014 study of SPC counts [17], as the different guidelines take different approaches to coding SPCs in paired organs [20]. However, the same study [17] found non-breast SPC counts to be almost identical under either set of guidelines. Therefore, only non-breast cancers were considered as SPCs in this review.

The chosen measure of association was the standardised incidence ratio (SIR), which compares the incidence of non-breast SPCs among men with a prior first primary BC to the corresponding expected incidence of non-breast primaries in the general male population.

### Data sources and search strategy

PubMed, Embase and Web of Science were each searched for relevant studies on March 11, 2022, using queries described in the Supplementary Material.

### Inclusion and exclusion criteria

Studies were considered for inclusion if a SIR and associated standard error could be extracted that assessed the combined risk of non-breast SPCs in male BC survivors, they focussed primarily on adults and they were written in English. A final inclusion criterion was that a study must use IARC/IACR or SEER rules to identify SPCs, or if this was unstated, must state that diagnoses of any SPCs had been confirmed by a physician, with efforts made to differentiate from recurrences or metastases. Studies were excluded if they reported solely on SPC risks following a specific treatment (or lack thereof) of the first male BC, they reported solely on SPC risks following a non-invasive first male BC, or they had a cohort of fewer than 100 male BC survivors.

Studies with data overlapping entirely with another study were also excluded. Partially overlapping studies were included in the SR, although only the larger study was included in any meta-analyses. Data from the Swedish Family Cancer Database were considered to overlap with data from the Swedish national cancer registry due to close links between these resources [21].

### Data extraction

Titles and abstracts were screened independently by two authors, with a third author resolving any conflicts. For each study, the first author, publication date, country and centre of data derivation, design, time period, follow-up and definitions of the cohort and of SPCs were extracted, together with additional fields such as stratification details and sample sizes. One author was contacted for clarification. The extracted data were input into a Microsoft Excel table.

### Statistical analysis

All statistical analyses were performed in R version 4.1.2 [22]. For each eligible study, the SIR of developing any non-breast SPC following an invasive first primary male BC was extracted as the principal summary measure. Meta-analyses were performed using the random-effects generic inverse-variance method, with DerSimonian–Laird estimators [23, 24]. Standard errors were extracted by dividing the square root of observed non-breast SPC counts by the corresponding expected counts and were converted to the natural logarithm scale by dividing the result by the corresponding SIR [25]. When unreported, expected SPC counts were estimated by dividing observed SPC counts by SIRs. Unreported confidence intervals (CIs) were estimated using Byar’s approximation, assuming observed SPC counts followed a Poisson distribution [25].

We performed unstratified meta-analyses and also stratified by age and time elapsed since the onset of the first BC. The stratification point for age was set at 50 years, although data on men aged up to 60 at BC onset were added into the younger group if no stratification at 50 was provided. We also performed two separate meta-analyses, respectively stratifying at 5 years and 10 years post diagnosis of the first BC. We considered reported SIRs stratified at 9 years equivalent to reported SIRs stratified at 10 years.

We also performed sixteen further meta-analyses, respectively evaluating second cancer risks at the following specific sites: bladder, blood (leukaemia, myeloma and non-Hodgkins lymphoma), brain and central nervous system (CNS), colorectum, head and neck, kidney, liver, lung, oesophagus, pancreas, prostate, skin (melanoma), stomach and thyroid. These are the male-specific subset of the 20 most common cancer sites in the UK from 2016 to 2018, after excluding BC and cancer of unknown primaries [26]. Since the purpose was to examine the distribution of the sites of any observed combined SPC risks, a study providing a SIR and associated standard error of developing cancer at a specific site was included in the corresponding site-specific meta-analysis only if it was also included in the meta-analysis that was unrestricted by SPC site.

We assessed between-study heterogeneity using Cochran’s Q [27] and the I^2^ statistic [28, 29]. Publication bias was assessed using funnel plots and Egger’s test [30]. A study was regarded as an outlier if there was no overlap between the study-specific and pooled (unstratified) meta-analysis confidence intervals [31]. All meta-analyses were performed first, including, then excluding, outlier studies. The results of all meta-analyses were visually represented as forest plots. Further sensitivity analyses took the form of subgroup analyses testing the effect of the geographical region (continent) of data derivation. Differences between summary SIRs based on multiple different datasets, such as different age groups, were assessed by treating each set of data as a subgroup and comparing the resulting Cochran’s Q to a chi-squared distribution with the degrees of freedom being the number of subgroups minus one [31]. *P* values of less than 0.05 were deemed significant.

Study quality was assessed using the Newcastle–Ottawa scale (NOS) [32] (details in Supplementary Material).

## Results

### Results of literature search

**Fig. 1:**
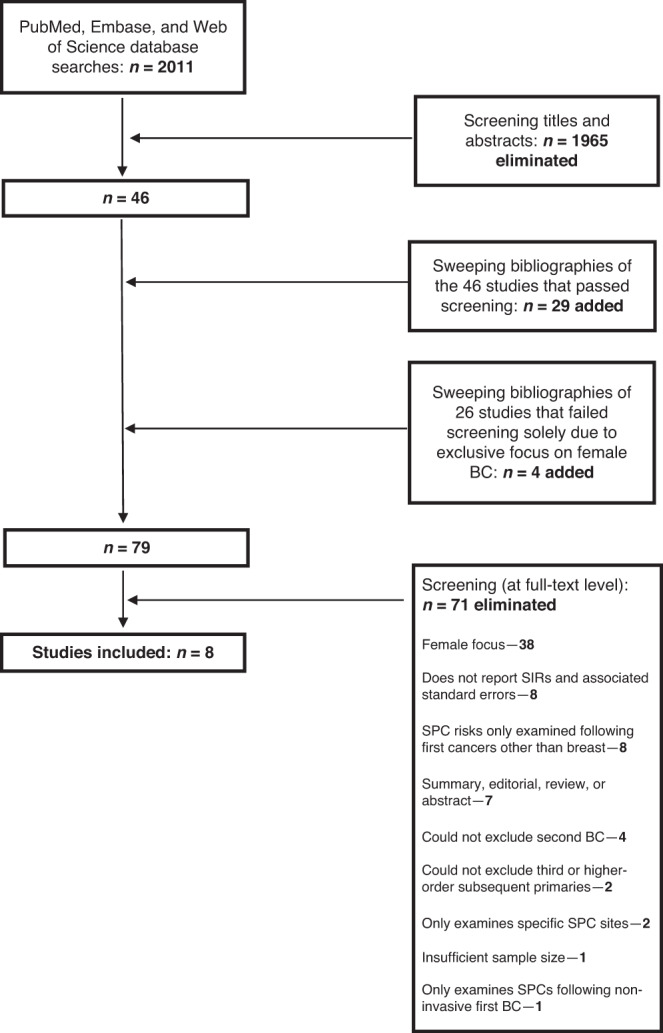
Search process. The search process used to identify the studies in this review, as described in the Methods section.

The database searches yielded 2011 studies following deduplication, 46 of which were deemed suitable for full-text screening as well as bibliography sweeping. To ensure the capture of all relevant studies, we also swept the bibliographies of 26 studies deemed unsuitable for full-text screening solely due to their focus on female BC survivors. Overall, the bibliography sweeps yielded 33 additional studies for full-text screening. In total, eight studies were included in the SR (Fig. 1).

The total number of male BC survivors among the six studies [5–9, 12] which reported sample sizes was 10,038. All studies reported the number of SPCs which developed following male BC, yielding a total of 1451. Six studies [4, 6–9, 12] reported the total follow-up time contributed by their cohort, totalling 36,315 person-years.

All studies were population-based and followed a retrospective cohort design, with follow-up periods lasting between 63 [5] and 13 years [3]. The reported SIRs ranged from 1.05 [8] to 2.17 [7], with the majority lying between 1.05 and 1.34 [3–6, 8, 9, 12].

Further study characteristics are described in Tables 1 and 2. NOS scores may be seen in the Supplementary Material.

**Table 1:** Study characteristics.

Author and publication year	Period of first BC dx^a^ for cohort/end of follow-up (if different)	Study design	Country and centre of data derivation	Definition of cohort	Definition of SPCs^b^
AIRTUM Working Group, 2013 [6]	Dx 1976–2010	Retrospective cohort	Italy (multiple cancer registries covering up to 48% of the population)	All patients dx with a first cancer, although melanoma skin cancer cases, cases based on death certificate only, cases based on autopsy only, and cases with follow-up time equal to zero were excluded. Cohort was stratified by first cancer site, allowing analysis for first BC.	According to IARC/IACR^c^ rules
Chen, 2015 [3]	Dx 1997–2010	Retrospective cohort	Sweden (FCD^d^) and Germany (12 German cancer registries covering 33% of the population)	Patients aged 15 y^e^ or over at dx of a first primary malignant tumour. Patients with only death certificate/autopsy information were excluded. Cohort was stratified by first cancer site, allowing analysis for first BC.	Germany: According to IARC/IACR rules, not including non-melanoma skin cancer. Sweden: SPC coding rules unstated, but Swedish FCD is linked to the national registry, which uses IARC/IACR rules. Malignancies had to be “clearly separated” to be registered as multiple primaries.
Dong, 2001 [9]	Dx 1958–1996	Retrospective cohort	Sweden—FCD	All patients dx with an invasive cancer as a first primary malignancy that was reported to the Swedish FCD. Cohort was stratified by first cancer site, allowing analysis for first BC.	SPC coding rules unstated, but Swedish FCD is linked to Swedish national cancer registry, which uses IARC/AICR rules.
Hemminki, 2005 [5]	All Dx; Australia, New South Wales: 1972–1997, Canada, British Colombia: 1970–1998, Canada, Manitoba: 1970–1998, Canada, Saskatchewan: 1967–1998, Denmark: 1943–1997, Finland: 1953–1998, Iceland: 1955–2000, Norway: 1953–1999, Singapore, Chinese: 1968–1992, Slovenia: 1961–1998, Spain, Zaragoza: 1978–1998, Sweden: 1961–1998, UK, Scotland: 1975–1996	Retrospective cohort	13 large cancer registries. Canada (British Columbia, Manitoba and Saskatchewan), Singapore, Slovenia, Norway, Denmark, Scotland, Australia (New South Wales), Sweden, Finland, Iceland, Spain (Zaragoza)	Men dx with a first BC.	According to IARC/IACR rules. Tumours recorded according to the practice of the participating centres.
Hung, 2016 [7]	Dx 1997–2010, follow-up until 2011	Retrospective cohort	Taiwan (Registry of Catastrophic Illness)	Patients dx with a first BC.	SPC coding rules unstated, but the registry histologically confirms cancer cases, and oncologists are required to give evidence of the diagnosis for review by commissioned expert panels. This evidence could include cytology reports, pathology reports, laboratory studies, and imaging studies.
Jégu, 2014 [4]	Dx 1989–2004, follow-up until 2007	Retrospective cohort	France (10 registries covering the Bas-Rhin, Calvados, Doubs, Hérault, Isère, Manche, Somme and Tarn administrative regions)	Patients dx with a first cancer. Cohort was stratified by first cancer site, allowing analysis for first BC.	According to IARC/IACR rules, with second primary cancers occurring at least 2 m^f^ (≥61 days) after a first cancer.
Satram-Hoang, 2007 [8]	Dx 1988–2003	Retrospective cohort	USA—California Cancer Registry	Men aged under 85 dx with first primary BC, registered at California Cancer Registry.	According to SEER^g^ rules. Accepted SPCs had to be malignant, metachronous, and develop at least 2 m post-BC dx. Synchronous SPCs developing before this were excluded.
Sung, 2020 [12]	Dx 1992–2011, follow-up until 2017	Retrospective cohort	USA—12 large cancer registries covering 13% of the USA population (Atlanta (Metropolitan), Connecticut, Detroit (Metropolitan), Hawaii, Iowa, Los Angeles, New Mexico, Rural Georgia, San Francisco (Oakland), San Jose (Monterey), Seattle (Puget Sound), Utah)	Patients aged 20–84 dx with a first primary malignant cancer, who had survived at least 5 years since dx. Cohort was stratified by the first cancer site, allowing analysis for first BC.	According to SEER rules.

*BC* breast cancer.

^a^Diagnosis/diagnoses/diagnosed.

^b^Second primary cancer.

^c^International Association of Cancer Registries/International Agency for Research on Cancer.

^d^Family cancer database.

^e^Year/years.

^f^Month/months.

^g^Surveillance, epidemiology and end results.

**Table 2:** Further study characteristics and standardised incidence ratio estimates.

Author and publication year	Total person-years	Follow-up time strata (since first breast cancer diagnosis)	Age strata (at first breast cancer diagnosis)	Specific second primary cancers for which standardised incidence ratios were reported	Number with first breast cancer/number with second primary cancer	Standardised incidence ratio (95% confidence interval) for combined risk of non-breast second primary cancers
AIRTUM Working Group, 2013 [6]	9402	0–1 m^a^, 2–11 m, 12–59 m, 60–119 m, >120 m	0–19, 20–29, 30–39, 40–49, 50–69, >70	Bladder and urinary tract, bone, brain and central nervous system, colon, colon rectum, gallbladder, head and neck, Hodgkin lymphoma, Kaposi sarcoma, kidney and renal pelvis, larynx, leukaemias, liver, lung, lymphoid leukaemia, mesothylioma, multiple myeloma, myeloid leukaemia, non-Hodgkin lymphomas, oesophagus, oral cavity, other leukaemias, other sites, pancreas, pharynx, prostate, rectum, skin melanoma, soft tissue, stomach, testis, thyroid, urinary bladder, urinary tract	1904/221	1.11 (0.97–1.27)
Chen, 2015 [3]	Germany: unreported Sweden: unreported	Germany: unreported Sweden: unreported	Germany: unreported Sweden: unreported	Germany: unreported Sweden: unreported	Germany: unreported/104 Sweden: unreported/52	Germany: 1.1 (0.9–1.3) Sweden: 1.1 (0.8–1.5)
Dong, 2001 [9]	3105	0–9 y^b^, 10–38 y	Unreported	Unreported	457/50	1.22 (0.91–1.61)^c^
Hemminki, 2005 [5]	Unreported	<1 y, 1–9 y, >9 y	<56, 56–65, 66–74, >75	Oral cavity and pharynx, stomach, small intestine, colorectal, colon, rectum, liver (both alone and including gallbladder and bile ducts), pancreas, larynx, lung, melanoma of skin, other neoplasm of skin, prostate, bladder, kidney, lymphohaematopoietic (all lymphomas combined, non-Hodgkins lymphoma, multiple myeloma, and leukaemias (lymphoid leukaemia, myeloid leukaemia))	3409/426	1.34 (1.22–1.47)
Hung, 2016 [7]	2773	0–1 y, 1–5 y, >=4 y	20–29, 30–39, 40–49, 50–59, 60–69, 70–79, >80	Head and neck, oesophagus, stomach, colon and rectum and anus, liver and biliary tract, liver, lung and mediastinum, bone and soft tissue, skin, prostate, bladder, kidney, thyroid, haematologic malignancies, all others	578/73	2.17 (1.70–2.73)
Jégu, 2014 [4]	2282	Unreported	Unreported	Unreported	Unreported/52	1.14 (0.85–1.50)^c^
Satram-Hoang, 2007 [8]	8529	<1 y, 1–5 y, >5 y	<60 y, 60–69 y, >69 y	Prostate, colorectal, lung and bronchus, bladder, melanoma, stomach	1986/201	1.05 (0.91–1.20)
Sung, 2020 [12]	10224	Unreported	Unreported	Unreported	1704/272	1.14 (1–1.3)

^a^Month/months.

^b^Year/years.

^c^Confidence interval generated using Byar’s approximation.

### Results of meta-analyses

To aid the interpretation of the results of the meta-analyses, it should be noted that one study reported two sets of SIRs, including and excluding data from the first 2 months of follow-up [6]. The latter results were described by the study as the more reliable and hence were used in the meta-analyses. In addition, one study pooled their data from multiple centres across four continents [5]. This study was regarded as European for any meta-analyses stratified by geographic region, since the bulk of their data was drawn from European registries.

#### Unstratified results

The unstratified meta-analysis included six studies [3–8]. Only the German subset of the data used by Chen et al. [3] was included due to the rest of the data partially overlapping with a much larger study [5]. All studies reported an increase in SPC risks following a first primary male BC. Some variation in the reported SIRs was present, with the largest studies reporting estimates between 1.05 and 1.34 [3–6, 8]. The only Asian study was an outlier, reporting a SIR of 2.17 [7]. There was no significant evidence for publication bias (Supplementary Material).

**Fig. 2:**
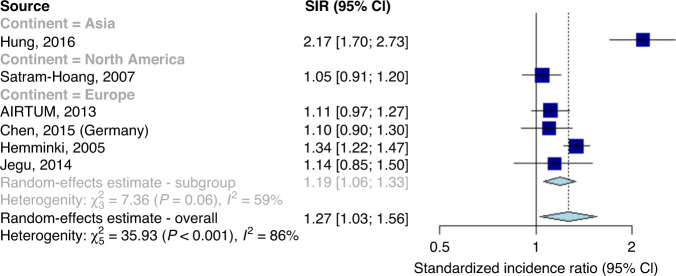
Forest plot showing standardised incidence ratios and a pooled estimate of second primary cancer risks. Association between a first primary male breast cancer and the onset of a non-breast second primary cancer, in comparison to the general male population, including the outlying study by Hung et al.

The summary SIR was estimated as 1.27 (95% CI: 1.03–1.56, Fig. 2). Significant heterogeneity was observed (Q: 35.93, *I*^2^: 86%, *P* < 0.001). Significant evidence was found for geographical location affecting summary SIRs (SIR: 2.17, 95% CI: 1.70–2.73 for the Asian study vs 1.19 (1.06–1.33) for European studies vs 1.05 (0.91–1.20) for the North American study, *P* for difference <0.001).

The study by Hung et al. reported a lower 95% CI bound of 1.70, which was greater than the upper 95% CI bound of 1.56 estimated in the above meta-analysis. Therefore, Hung et al. was regarded as an outlier, and thus all meta-analyses were performed twice: once including, and once excluding, Hung et al. No other outlier studies were present.

**Fig. 3:**
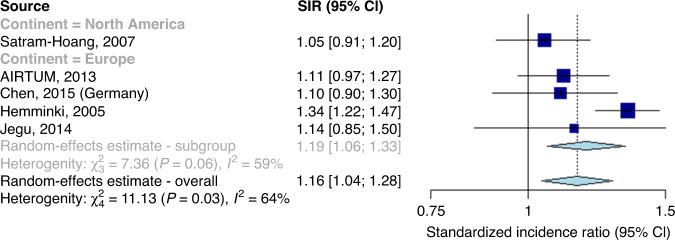
Forest plot showing standardised incidence ratios and a pooled estimate of second primary cancer risks. Association between a first primary male breast cancer and the onset of a non-breast second primary cancer, in comparison to the general male population, excluding the outlying study by Hung et al.

After excluding Hung et al., the summary SIR was estimated as 1.16 (95% CI: 1.04–1.28, Fig. 3). Heterogeneity decreased, but remained significant (Q: 11.13, *I*^2^: 64%, *P*: 0.025). There was no longer significant evidence for a difference in summary SIR by geographical location (Supplementary Material).

Whether including or excluding Hung et al., no significant evidence of heterogeneity was found within the continent-specific subgroups (Supplementary Material).

#### Effects of age at BC onset

The age-stratified meta-analyses consisted of 4 studies [5–8]. When including Hung et al., we found no significant evidence for a difference between the summary SIR of men aged under 50 at first BC onset and the summary SIR of men aged over 50 at first BC onset (Supplementary Material).

**Fig. 4:**
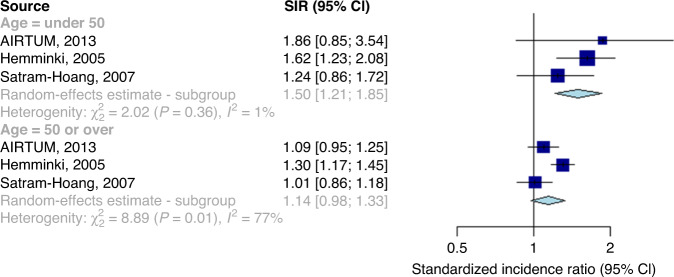
Forest plot showing standardised incidence ratios and a pooled estimate of second primary cancer risks, stratified by age group at breast cancer onset. Association between a first primary male breast cancer and the onset of a non-breast second primary cancer, in comparison to the general male population, stratified by age group at breast cancer onset, excluding the outlying study by Hung et al.

Results when excluding Hung et al. are shown in Fig. 4. There was significant evidence for a difference in summary SIR between the age groups (SIR: 1.50, 95% CI: 1.21–1.85) for those aged under 50 at first BC onset vs. 1.14 (95% CI: 0.98–1.33) for those aged over 50 at first BC onset, *P* for difference: 0.040).

#### Effects of follow-up time elapsed since BC onset

We found no significant evidence for the length of time elapsed since the onset of the first BC affecting SPC risks (Supplementary Material).

#### Site-specific associations

Hung et al. provided sufficient data for inclusion in the meta-analyses assessing the risks of SPCs at ten of the examined sites —the bladder, colorectum, head and neck, kidney, liver, lung, pancreas, prostate, stomach and thyroid. Summary SIRs from these meta-analyses ranged from 1.09 to 5.58. Among these sites, the risks of second primaries were significantly higher than the risks for first primaries for colorectal cancer (SIR: 1.29, 95% CI: 1.03–1.61), pancreatic cancer (SIR: 1.64, 95% CI: 1.05–2.55) and thyroid cancer (5.58, 95% CI: 1.04–30.05). Following the exclusion of Hung et al., there was no significant evidence of elevated cancer risks following male BC for any of these ten sites other than the colorectum (SIR: 1.21, 95% CI: 1.00–1.46)), although all associated point estimates were greater than 1. Hung et al. did not provide sufficient data for inclusion in the meta-analyses of SPC risks at the remaining examined sites—the blood (leukaemia, myeloma and non-Hodgkins lymphoma), the brain and CNS, the oesophagus, and the skin (melanoma). The summary SIRs generated for these sites ranged from 1.00 for the blood (non-Hodgkins lymphoma) to 1.65 for the skin (melanoma), with no significant evidence for an increased risk of second primaries at any of these six sites.

Full results may be seen in Supplementary Material.

## Discussion

Most published studies reporting SPC risks following male BC draw their data from European [3, 4, 6, 9] or North American [8, 10, 12] population-based cancer registries. The majority reported elevated risks [3–9, 12]. Male BC survivors have often been found to be at greater risk of primary cancers of the prostate [5–7, 10], skin [5, 7, 8, 10] and digestive system [5–8], although with varying magnitudes.

This systematic review and meta-analysis of such studies confirm the combined risks of non-breast SPCs to be significantly elevated following a first primary male BC. When excluding the outlier study by Hung et al., male BC survivors aged under 50 at the initial BC diagnosis were found to be at significantly higher risk than those over 50. This difference in risks may even have been slightly underestimated, due to our decision to include data on men aged under 60 at first BC diagnosis in the younger stratum when no direct stratification at 50 was provided [33]. We also found significant differences between risks reported by studies from Asia, North America and Europe, although larger studies from a wider range of countries are needed to clarify the extent of any risk differences between geographic regions. Finally, we found that male BC survivors are at increased risk of second colorectal, pancreatic and thyroid cancer.

The results of the age-stratified, continent-stratified and site-specific meta-analyses differed depending on whether Hung et al. were included. We therefore discuss the robustness of these results here. Firstly, we found significant evidence for SPC risks varying by geographical region only when including Hung et al. Since Hung et al. was the sole Asian study, this indicates that the difference was driven by this study rather than differences between the European and North American studies. Hung et al. is a well-designed study, using a complete and accurate database [34]. It therefore seems this finding may reflect a higher SPC risk in Asian (specifically Taiwanese) male BC survivors than a flaw in study design.

Although when the Hung et al. study was included in the meta-analysis, there was no significant difference in the SIRs by age at diagnosis, Hung et al. themselves found that men aged under 50 at the first BC diagnosis were at substantially greater SPC risk than men aged over 50 (SIR: 5.68, 95% CI: 1.83–13.26) for those under 50 vs 2.08 (1.61–2.63) for those over 50, p for difference: 0.030). Therefore, the patterns of younger male BC survivors being at greater SPC risk than older male BC survivors, which were seen when Hung et al. was excluded, are consistent.

Significant evidence for increased risks of second primary pancreatic and thyroid cancers was only found when Hung et al. was included in the relevant meta-analyses. The largest study in this review also found the risks of pancreatic SPCs to be significantly elevated [5]. There is also evidence of shared risk factors for male BC and pancreatic cancers. For example, pathogenic *BRCA1* [35, 36], *BRCA2* [35–37] and *PALB2* [37, 38] variants are associated with both male BC and pancreatic cancer. Therefore, the finding that male BC survivors are at increased pancreatic cancer risk seems plausible. In contrast, the finding of increased second primary thyroid cancer risks was mainly driven by data from Hung et al. and was based on a total of just 4 observed cases. Although previous BC has been linked to elevated thyroid cancer risks in women [39], larger studies are needed to clarify this association in men. Finally, it should be noted that Hung et al. reported combined risks of colorectal and anal cancers and of lung and mediastinum cancers. Hence, the point estimates estimated for second colorectal and lung cancers when data from Hung et al. were included may be distorted slightly, although the fact that second colorectal cancer risks remained significant even following the exclusion of these data indicates that second colorectal primary risk is likely to be elevated in male BC survivors.

The strengths of this SR include the number of studies with large sample sizes, considering the rarity of male BC [5, 6, 8, 12]. There was also no significant evidence of publication bias (see Supplementary Material). This SR was built on studies of high methodological quality, with all studies being assigned NOS scores of 6 or higher. Finally, there was limited heterogeneity among European studies, which was the largest continent-specific subset of studies available.

It is known that BC treatments such as chemotherapy, radiotherapy, or hormonal therapy increase SPC risks in women [40–42]. Treatment effects could also partly explain our findings in men. Other non-genetic risk factors which may influence risks for the first primary male BC, such as hormonal imbalances or a family history of male BC [1], may also contribute to the observed elevated SIRs. However, this information was not available in the studies. Notably, in addition to pancreatic cancer, some cancers found to be at greater risk following male BC are also associated with pathogenic variants in genes linked to BC susceptibility in men. For example, both male BC susceptibility [37, 43] and colorectal cancer susceptibility [44, 45] are associated with pathogenic variants in the *CHEK2* gene. We also found some evidence of elevated second stomach and prostate cancer risks when including Hung et al., although the associations were not significant (Prostate cancer: SIR: 1.32, 95% CI: 1.00–1.76, *P*: 0.050. Stomach cancer: SIR: 1.35, 95% CI: 0.99–1.84, *P*: 0.058). Both cancers are also associated with pathogenic variants in male BC susceptibility genes: prostate cancer with the *BRCA1/2* [35, 36, 46] and *CHEK2* [47–49] genes and stomach cancer with the *BRCA1/2* [35, 36, 50] and *CHEK2* [51] genes.

This evidence suggests that SPC risks for BC survivors with a genetic predisposition to BC may be increased in comparison to BC survivors without such a predisposition. Research in this area has been undertaken for contralateral BC in women [52], but is otherwise very scarce. There is some evidence that a higher proportion of male than female BC cases are due to pathogenic variants in BC susceptibility genes [53, 54], with the largest study of germline susceptibility in male BC cases finding 13.7% of male BC survivors to carry such variants [37]. Pathogenic germline variants in BC susceptibility genes could account for a sizeable proportion of second primaries following male BC, with a recent large study confirming non-breast primaries to be 58% more common among male carriers of deleterious *BRCA1/2* variants than among male relatives of carriers who were either untested for, or confirmed not to carry, such a variant [35]. Further research in this area may thus be particularly relevant for male BC survivors. Genetic susceptibility could also account for part of the observed association between early-onset male BC and raised SPC risks, since pathogenic variants in such genes are associated with an earlier age at BC diagnosis [55–57]. An additional explanation for this relationship is that more aggressive treatment regimens tend to be offered to younger BC patients [58, 59], but these treatments can confer a higher risk of developing SPCs [40–42].

The study has some limitations. The estimated SIRs may have been affected by surveillance bias, whereby cancers are detected in BC survivors that would have gone unnoticed in individuals without any cancer history due to increased surveillance [6, 60]. However, this was likely reduced by the inclusion of data from four studies [4, 6, 8, 12] excluding SPCs occurring within a time period of at least 2 months immediately following the initial BC diagnosis, where surveillance bias is likely to be most intensive [6]. The paucity of studies reporting effects of treatments of the first male BC [7, 8] and the lack of studies reporting the influence of hormonal imbalances and family histories of male BC also meant that we could not adjust for several potential confounders. The rarity of second cancers at certain sites may also mean some analyses were underpowered, as evidenced by the wide confidence intervals. Therefore, it cannot be concluded that other associations do not exist. It also cannot be ruled out that some relevant published studies were missed, although the double-screening process and the sweeps of reference lists should minimise the likelihood of this.

To our knowledge, this is the first meta-analysis of SPC risks in male BC survivors to have been performed and the first systematic review since 2008. This study provided site-specific SIRs and assessed the variability in the estimates by age at first BC diagnosis, follow-up time and geographical region (continent). Future large cohort studies might consider the effects of BC treatment, family history, or hormonal imbalances, as they receive relatively little focus in the current literature. There is also a clear need for further research on the influence of pathogenic variants in BC susceptibility genes on SPC risks following male BC.

### Reporting summary

Further information on research design is available in the Nature Research Reporting Summary linked to this article.

## Supplementary information

Supplementary Material

PRISMA checklist for abstract

PRISMA checklist for manuscript

Reporting summary - completed checklist

## Data Availability

The data from the study by the AIRTUM Working Group are publicly available at I tumori in Italia. Rapporto 2013: I tumori multipli | Epidemiologia&Prevenzione (epiprev.it). All data from the remaining studies were taken directly from their corresponding published, publicly available manuscripts or Supplementary Materials.

## Code availability

All codes used to generate the results in this manuscript can be provided upon request.
