# Supplementary material for: Risk of developing a second primary cancer in male breast cancer survivors: a systematic review and meta-analysis
Source: Br J Cancer. 2022 Sep 17;127(9):1660–9. doi: 10.1038/s41416-022-01940-1 (PMC9596702; doi:10.1038/s41416-022-01940-1)
Supplement: Supplementary file 1 — Supplementary Material [file 41416_2022_1940_MOESM1_ESM.docx]

Supplementary Material

This supplementary material contains an exact description of the queries used to search the PubMed, Embase and Web Of Science databases for relevant studies. It also shows extended results for the meta-analyses stratified by geographic region, by age at the onset of the first male BC and by time elapsed since the onset of the first male BC. It lastly presents extended results for the site-specific meta-analyses, the methods used in and the results of the assessments of study quality, and an assessment of publication bias.

# Search strategy

Below are the queries used to search PubMed, Embase, and Web of Science for relevant studies on the 11^th^ of March 2022.

**PubMed query:**

*("Breast Neoplasms"[MeSH] OR "breast cancer") AND ("Neoplasms, Second Primary"[MeSH] OR "second cancer" OR "second primary") AND risk*

**Embase query:**

*(Breast Neoplasms/ or "breast cancer") and (Neoplasms, Second Primary/ or "second cancer" or "second primary") and risk*

**Web of Science query:**

*(TS = (("breast cancer" OR "breast neoplasm") AND ("second cancer" or "second primary") AND risk)) OR (AB = (("breast cancer" OR "breast neoplasm") AND ("second cancer" or "second primary") AND risk))*

# Effects of geographic region:

There was no significant evidence for a difference in summary SIRs between geographic regions when Hung et al. was excluded (SIR: 1.19, 95%CI: 1.06-1.33) for European studies vs 1.05 (95%CI: 0.91-1.20) for the North American study, p for difference: 0.18, Figure 2, main article). Results when including Hung et al. are described in the main article.

We found no significant evidence of heterogeneity in any of the region-specific subgroups (Q: 7.36, I^2^: 59%, p: 0.061 for European studies. Heterogeneity could not be evaluated for the remaining subgroup(s) as they all consisted of one study (Figure 2, main article).

#

# Effects of age at BC onset:

Results when including the outlying study by Hung et al. may be seen in Figure S1 (results when excluding Hung et al. may be seen in Figure 4, cited in main article). There was no significant evidence found for a difference in summary SIR between the age groups (SIR: 1.93 (95% CI: 1.12-3.31) for men aged under 50 at first BC onset vs. 1.30 (0.96-1.76) for men aged over 50 at first BC onset, p for difference: 0.212).

**Figure S1: Association between a first primary male breast cancer and the onset of a non-breast second primary cancer, in comparison to the general male population, stratified by age group at breast cancer onset, including the outlying study by Hung et al.**

**
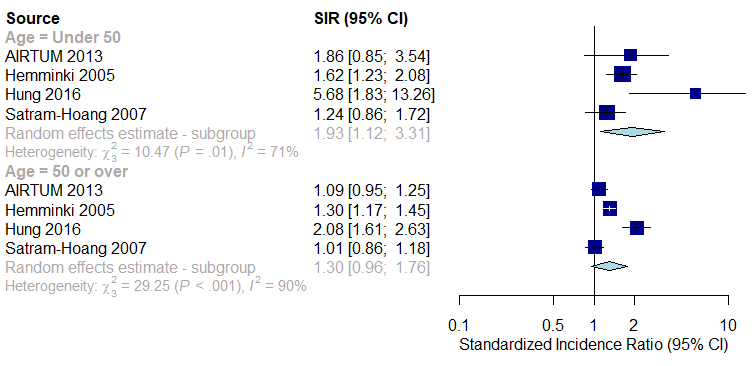
**

# Effects of follow-up time elapsed since BC onset:

The meta-analysis which stratified at 5 years elapsed since male BC onset consisted of 3 studies^6-8^. As shown in Figure S2, no significant evidence for a difference in summary SIR between strata was observed (SIR: 1.27 (95%CI: 0.66-2.46) below 5-year point vs 1.33 (95%CI: 0.98-1.80) above 5-year point, p for difference = 0.912).


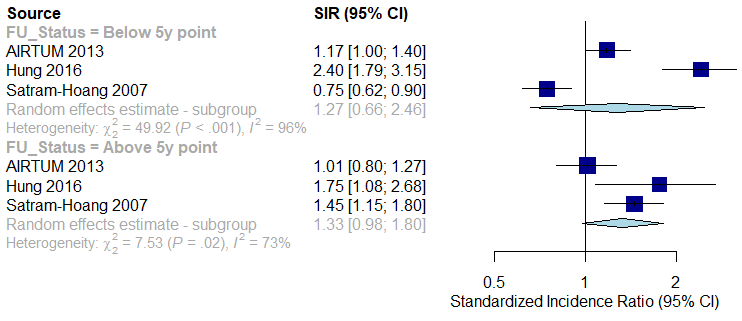
**Figure S2: Association between a first primary male breast cancer and the onset of a non-breast second primary cancer, in comparison to the general male population, stratified by time elapsed since breast cancer onset, with stratification point set at 5 years, including the outlying study by Hung et al.**

The results following the elimination of Hung et al. may be seen in Figure S3. No significant evidence for a difference in summary SIR between strata was observed (SIR: 0.94 (95%CI: 0.60-1.46) below 5-year point vs 1.21 (95%CI: 0.86-1.72) above 5-year point, p for difference = 0.368).

**
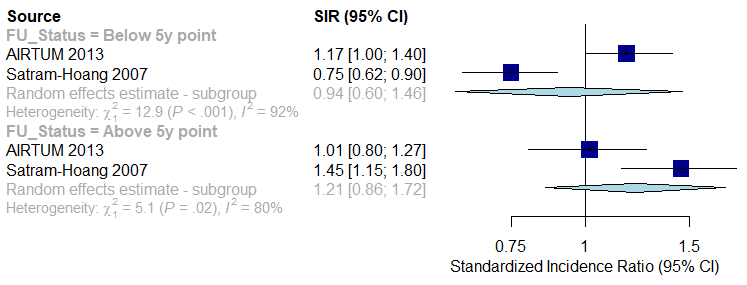
Figure S3: Association between a first primary male breast cancer and the onset of a non-breast second primary cancer, in comparison to the general male population, stratified by time elapsed since breast cancer onset, with stratification point set at 5 years, excluding the outlying study by Hung et al.**

The meta-analysis dividing the groups at 10 years elapsed since BC onset consisted of 2 studies^5-6^. As shown in Figure S4, there was no significant evidence for a difference in summary SIR (SIR: 1.27 (95%CI: 1.07-1.50) below the 10-year point vs 1.08 (0.74-1.57) above the 10-year point, p for difference = 0.433).


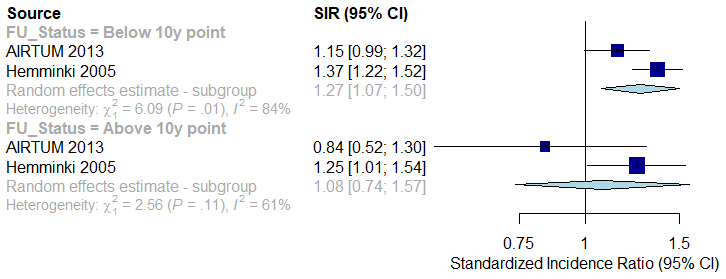
**Figure S4: Association between a first primary male breast cancer and the onset of a non-breast second primary cancer, in comparison to the general male population, stratified by time elapsed since breast cancer onset, with stratification point set at 10 years.**

# Site-specific associations:

Full results of the meta-analyses for the sixteen examined sites may be seen in Table S1. Results are presented both including and excluding data from Hung et al. for the bladder, head and neck, kidney, liver, pancreas, prostate, stomach, and thyroid. Hung was excluded from the meta-analyses concerning all other sites, as relevant data was not provided. Significant results are highlighted in bold.

**Table S1: Results of meta-analyses examining site-specific SPCs**

| **Second primary cancer site** | **Summary SIR (95% CI), including Hung et al.** | **Summary SIR (95% CI), excluding Hung et al.** | **Number of studies in meta-analysis, including Hung et al.** | **Total number of site-specific SPCs recorded, including Hung et al.** |
| --- | --- | --- | --- | --- |
| Bladder | 1.14 (0.85 - 1.52) | 1.11 (0.81-1.51) | 4^5-8^ | 63 |
| Blood (Leukaemia) | Hung et al. not included | 1.13 (0.24 - 5.37) | 2^5-6^ | 19 |
| Blood (Myeloma) | Hung et al. not included | 1.02 (0.48 - 2.15) | 2^5-6^ | 6 |
| Blood (Non-Hodgkins Lymphoma) | Hung et al. not included | 1.00 (0.39 - 2.58) | 2^5-6^ | 14 |
| Brain and CNS | Hung et al. not included | 1.37 (0.28 - 4.01) | 1^6^ | 3 |
| Colorectum [1] | **1.29 (1.03-1.61)** | **1.21 (1.00-1.46)** | 3^5, 6, 8^ | 109 |
| Head and neck | 2.61 (0.93-7.33) | 1.54 (0.79-2.68) | 2^6-7^ | 25 |
| Kidney [2] | 1.44 (0.66 - 3.13) | 1.48 (0.54-4.09) | 3^5-7^ | 22 |
| Liver [3] | 1.20 (0.77 - 1.89) | 1.26 (0.62-2.59) | 3^5-7^ | 19 |
| Lung [4] | 1.09 (0.76-1.56) | 0.98 (0.70-1.36) | 3^5, 6, 8^ | 119 |
| Oesophagus | Hung et al. not included | 1.45 (0.29 - 4.24) | 1^6^ | 3 |
| Pancreas | **1.64 (1.05 - 2.55)** | 1.62 (0.98-2.68) | 3^5-7^ | 25 |
| Prostate | 1.32 (1.00-1.76) | 1.27 (0.93-1.72) | 4^5-8^ | 247 |
| Skin (Melanoma) | Hung et al. not included | 1.65 (0.77 - 3.54) | 3^5, 6, 8^ | 25 |
| Stomach | 1.34 (0.99 - 1.84) | 1.33 (0.94-1.89) | 4^5-8^ | 51 |
| Thyroid | **5.58 (1.04 - 30.05)** | 2.37 (0.27-8.55) | 2^6-7^ | 4 |
| 1: Second anal cancer cancers also included.  2: Second renal pelvis cancers also included.  3: Second biliary tract cancers also included.  4: Second bronchus and mediastinum cancers also included. | | |  |  |

# Assessments of study quality: Newcastle-Ottawa scale

The Newcastle-Ottawa scale (NOS) assesses each study in the categories of “selection”, “comparability”, and “outcome”, in which a maximum of 4, 2, and 3 stars can respectively be scored. A higher score is intended to translate to a lower risk of within-study bias. Isaac Allen assigned the NOS scores for each study.

## Methods:

NOS scores were calculating using the following criteria:

For a study to score 4 points in the NOS selection category, the cohort of male BC survivors must be representative of the average male BC survivor in the relevant community, the expected number of cancers in men without any cancer history must be calculated using the same data source the male BC survivors were drawn from, the first male BC diagnosis must be ascertained from secure records such as those from a registry or a structured interview of the subjects, and cancers other than non-melanoma skin cancer must not have been present prior to the first male BC^31^. To score 2 points for comparability, a study must control for age and calendar period at initial BC diagnosis. To score 3 points in the outcome category, a study must assess SPC development by record linkage or blind independent assessment, allow a follow-up period of at least 20 years, and have lost less than 10% of the initial cohort to follow-up.

## Results:

The breakdown of assigned scores on the Newcastle-Ottawa scale for each study featured in this systematic review may be seen in Table S2.

Sung et al.^12^ lost one star for selection as their cohort included only those who survived at least 5 years after their initial cancer diagnosis, which we deemed insufficiently representative of the average cancer survivor. All studies losing at least one star for selection did so due to the expected cancer rates either being derived from slightly different sources than the participating centres, such as a different closely linked registry or a superset of the participating centres^4, 7, 8^.

All studies other than Sung et al.^12^ lost a star in the outcome category due to leaving the proportion of the cohort lost to follow-up unstated. All studies which lost a further star did so due to allowing less than 20 years of follow-up^3, 4, 7, 8^.

**Table S2 – selection, comparability, outcome, and total scores on the Newcastle-Ottawa scale, for each study in the systematic review**

| **Author and publication year** | **NOS scale selection score (/4)** | **NOS scale comparability score (/2)** | **NOS scale outcome score (/3)** | **NOS scale total score (/9)** |
| --- | --- | --- | --- | --- |
| AIRTUM Working Group 2013^6^ | ★★★★ | ★★ | ★★ | 8 |
| Chen 2015^3^ | ★★★★ | ★★ | ★ | 7 |
| Dong 2001^9^ | ★★★★ | ★★ | ★★ | 8 |
| Hemminiki 2005^5^ | ★★★★ | ★★ | ★★ | 8 |
| Hung 2016^7^ | ★★★ | ★★ | ★ | 6 |
| Jégu 2014^4^ | ★★★ | ★★ | ★ | 6 |
| Satram-Hoang 2007^8^ | ★★★ | ★★ | ★ | 6 |
| Sung 2020^12^ | ★★★ | ★★ | ★★★ | 8 |

# Assessments of publication bias

Funnel plots and Egger’s test were used to evaluate and formally test for publication bias. As can be seen in Figure S5, the funnel plot does not indicate sizeable publication bias. Egger’s test yielded no significant evidence for publication bias (p = 0.781) although this should be interpreted with caution due to the small number of male studies^29^.


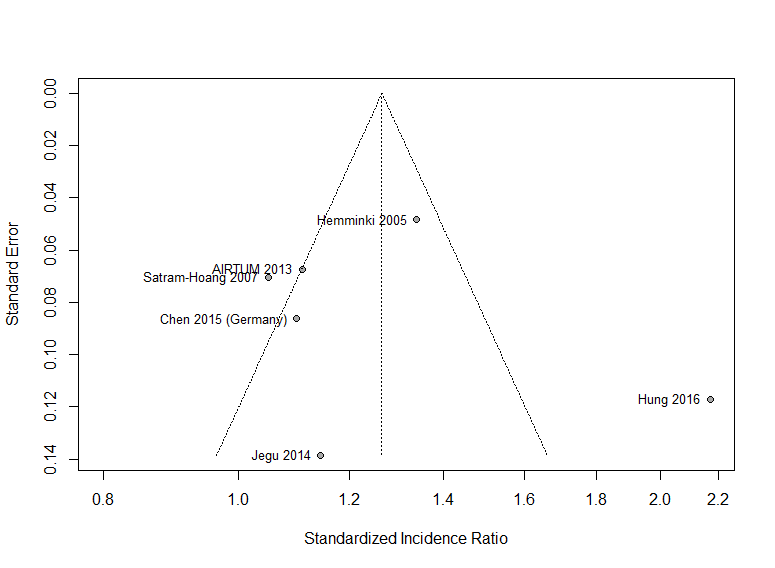
**Figure S5: Funnel plot of standardized incidence ratio against standard error, for all studies included in meta-analyses**

*Please note that all reference numbers and abbreviations in the supplementary material correspond to those in the main body of the article.*

*This review was not registered. No protocol was prepared.*
